# Prognostic value of neutrophil- lymphocyte count ratio (NLCR) among adult ICU patients in comparison to APACHE II score and conventional inflammatory markers: a multi center retrospective cohort study

**DOI:** 10.1186/s12873-021-00418-2

**Published:** 2021-02-23

**Authors:** Tao Zhou, Nan Zheng, Xiang Li, Dongmei Zhu, Yi Han

**Affiliations:** Department of Critical Care Medicine, the First Affiliated Hospital of Nanjing Medical University, Nanjing Medical University, 300 Guangzhou Road, Nanjing, 210029 China

**Keywords:** Neutrophil lymphocyte count ratio, Procalcitonin, CRP, APACHE II, Inflammation, Critical illness

## Abstract

**Background:**

Neutrophil-lymphocyte count ratio (NLCR) has been reported as better indicator of bacteremia than procalcitonin (PCT), and more precise predictor of mortality than C-reactive protein (CRP) under various medical conditions. However, large controversy remains upon this topic. To address the discrepancy, our group has compared the efficiency of NLCR with conventional inflammatory markers in predicting the prognosis of critical illness.

**Methods:**

We performed a multi-center retrospective cohort study involving 536 ICU patients with outcomes of survival, 28- and 7-day mortality. NLCR was compared with conventional inflammatory markers such as PCT, CRP, serum lactate (LAC), white blood cell, neutrophil and severity score APACHE II (Acute Physiology and Chronic Health Evaluation II) to evaluate the potential outcomes of critical illness. Then, receiver operating characteristics (ROC) curves were constructed to assess and compare each marker’s sensitivity and specificity respectively.

**Results:**

NLCR values were not different between survival and mortality groups. Meanwhile, remarkable differences were observed upon APACHE II score, CRP, PCT and LAC levels between survival and death groups. ROC analysis revealed that NLCR was not competent to predict prognosis of critical illness. The AUROCs of conventional markers such as CRP, PCT, LAC and APACHE II score were more effective in predicting 28- and 7-day mortality.

**Conclusions:**

NLCR is less reliable than conventional markers CRP, PCT, LAC and APACHE II score in assessing severity and in predicting outcomes of critical illness.

**Supplementary Information:**

The online version contains supplementary material available at 10.1186/s12873-021-00418-2.

## Background

Systemic inflammation is an integral part of pathophysiological processes in critical illness. The NLCR is a conveniently used marker that is readily calculated according to complete blood count [1]. Previously NLCR has been proved as a marker of infection, but did not obtain wide acceptance. Even though NLCR is a conveniently available marker, it did not obtain wide acceptance in clinical [2]. In contrast, cytokines and some acute phase proteins have been frequently used to assess the inflammatory processes in both clinical and research scenarios. C-reactive protein, white blood cell count and neutrophil percentage have long been recognized as valuable markers of inflammation [3]. Thus, these markers play great roles in recognition of inflammatory status, in assessing the severity of diseases and predicting the following outcomes. However, the sensitivity and specificity has yet to be determined.

The APACHE II scores are still widely accepted as the evaluation tools used for determining the criticality of critically ill patients and for evaluating their prognosis [4]. As in critically ill patients, especially in sepsis patients with infection, the number and proportion of neutrophils are elevated, whereas lymphocytes are decreased. Therefore, Zahorec et al. proposes that the neutrophil lymphocyte count ratio (NLCR) is a rapid, easy-applicable, and cost-effective parameter to evaluate the inflammatory and stress status of critically ill patients [1]. NLCR is biomarker based on proportion of neutrophil count in complete blood cell count, which increases in inflammatory disease. Conversely, lymphocytes usually reflect to the patient’s immune status and decrease as inflammatory disease worsens. Until recently, the diagnostic and prognostic values of NLCR were applied to myriad medical situations such as bacteremia [5], sepsis [6], myocardial infarction [7], aneurysmal subarachnoid hemorrhage [8], community acquired pneumonia [9], acute kidney injury [10], liver transplantation [11] and even colorectal cancer. It also provides a more reliable prediction of patient survival rates [12]. Currently, this remains a hot topic of an open discussion [13].

Systemic inflammation is an unavoidable process of critical disease, and its severity generally associated with the short- and long-term outcomes of critical patients [14]. A great many biomarkers such as CRP and PCT have been applied to assess the severity and progress of systemic inflammation in clinical and research scenarios [14] as well as to predict the prognosis of various diseases. Besides, lactate is another common biomarker to evaluate. However, these biomarkers may have limited use due to the lack of sensitivity and specificity as both infection and stress could lead to remarkable changes of these parameters.

Thus, the objective in our current research was to evaluate the association of NLCR with the outcomes of adult critical ill patients, and to determine whether such marker is superior than conventional biomarkers or not.

## Methods

### Study setting and data source

There are six intensive care units (ICU) wards in our hospital which is a tertiary university hospital (The First Affiliated Hospital of Nanjing Medical University),they are Integrated ICU, Geriatric Medicine ICU, Cardiothoracic ICU, Emergency ICU, general surgery ICU, Neurosurgery ICU. We conducted a retrospective study with data collected from these intensive care units. Each patient admitted to ICU has its own focus on diseases, and there are also some critically ill patients with overlapping diagnoses. All the data we extracted are blood tests immediately after entering ICU (including direct admission and transfer from other departments), exported from the hospitalization system to a spreadsheet and used for follow-up analysis. Patients included in this database were admitted in these ICUs from Jan 2018 to Jun 2019. All the physiological and pathophysiological data, microbiological results and survival outcomes were recorded accordingly. We have received ethical approval (2020-SR-055) from the institutional review boards (IRBs) at the First Affiliated Hospital of Nanjing Medical University. Since this study does not contain protected health information and all data were anonymously used, a waiver of the requirement for informed consent was approved by the IRBs.

### Patient records

Inclusion criteria of the patients: (1) Adult patients aged > 18 years; (2) Admitted to ICUs in the First Affiliated Hospital of Nanjing Medical University during the period from Jan 2018 to Jun 2019.

Exclusion criteria of the patients: (1) Hematological disease; (2) Chemotherapy; (3) Receiving glucocorticoids; (4) Receiving bone marrow stimulators.

We retrieved the following clinical information for each patient from the database at ICU admission, age, gender, diagnosis, APACHE II score, body temperature, white blood cell count, neutrophil percentage, blood lactate, PCT, CRP, microbiologic results, coexisting diseases and survival records. NLCR was calculated as the ratio of neutrophil and lymphocyte count, as previously described [1].

Patients were divided into three groups according to the survival records: (1) Survival group; (2) 28-day mortality group; (3) 7-day mortality group. The blood samples of the patients in the study were taken within 30 min after admission to the ICU. Generally, the patients admitted into ICUs were more or less on fluid administration, depending on individual circulatory conditions. The timing for blood taking was usually during the early stages of resuscitation.

### Statistical analysis

Statistical analysis and graph construction was performed using GraphPad Prism 5.0 and IBM SPSS Statistics 23. Descriptive analysis was conducted for all variables. One way-Anova was applied to evaluate the differences in NLCR, PCT, CRP, LAC levels and APACHE II scores among different groups. Receiver operating characteristic (ROC) curves were built to assess and compare the sensitivity and specificity of the NLCR, PCT, CRP, LAC and APACHE II score in predicting 28-day and 7-day mortality. The area under the ROC curves (AUROCs) varied from 0.5 to 1.0 were accepted, with higher values indicating increased discriminatory ability. Confidence intervals of AUROCs were calculated with non-parametric assumptions. Each biomarker’s discriminant ability was compared according to its individual AUROC. For all the comparison in this study, *P* < 0.05 was considered the difference to be statistically significant.

## Results

### General characteristics

**Fig. 1:**
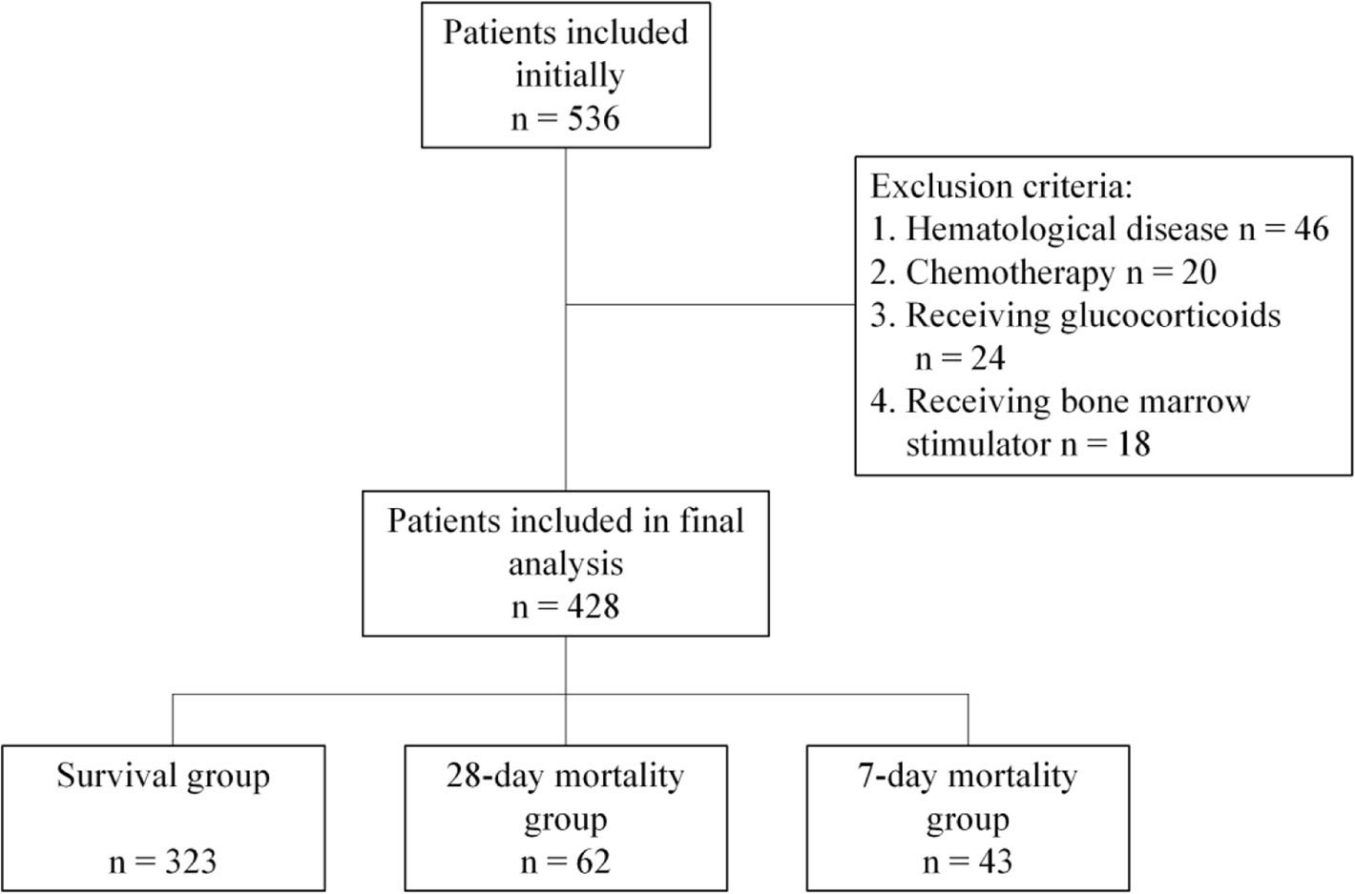
Enrollment flow chart

Initially, 536 patients were enrolled in this study. Following the flow chart with strict inclusion and exclusion criteria, data from 428 patients were finally analyzed in this study (Fig. 1), among which 310 were medical patients, and the rest were surgical patients. Overall, ICU mortality rate was 24.5% (105 of 428 patients), with 41.0% (43 of 105) deaths occurring during the first 7 days after admission, and 59% (62 of 105) deaths occurring between 7 and 28 days after admission. Table 1 shows the general characteristics of the total enrolled critical ill patients.

**Table 1:** Characteristics of the overall population

	Survival ***n*** = 323	28-day mortality ***n*** = 62	7-day mortality ***n*** = 43
**Age (years)**	67.0 ± 18.7	76.1 ± 12.7**	70.7 ± 16.9
**Sex (M/F)**	222/101	40/22	24/19
**Temperature abnormalities, n, (%)**	80 (24.8)	21 (33.3)	10 (23.3)
**WBC abnormalities, n, (%)**	112 (34.7)	24 (38.1)	25 (58.1)**
**NE abnormalities, n, (%)**	227 (70.3)	51 (82.3)	36 (83.7)
**APACHE II score**	18.8 ± 6.0	24.1 ± 5.4***	24.8 ± 7.8***
**NLCR**	11.5 ± 13.4	13.0 ± 11.3	15.0 ± 14.8
**PCT (ng/ml)**	3.6 ± 13.8	4.0 ± 12.5**	7.8 ± 15.0***
**CRP (mg/ml)**	61.1 ± 73.1	87.4 ± 69.1***	96.6 ± 76.0**
**Blood lactate (mmol/l)**	1.5 ± 1.0	1.9 ± 0.9**	2.7 ± 4.0
**Surgery, n, (%)**	99 (30.7)	16 (25.8)	3 (7.1)***
**Cardiovascular disease**	18 (5.6%)	4 (6.5%)	10 (23.3%)***
**Malignancies**	29 (9.0%)	1 (1.6%)**	4 (9.3%)

Data were expressed as number (percentage) of patients or mean ± standard deviation. **p < 0.01, ***p < 0.001 vs survival group. Temperature abnormality: temperature < 36 °C or > 38 °C; WBC abnormalities: WBC < 4 × 10 ^ 9 / L or > 12 × 10 ^ 9 / L; NE abnormality: neutrophil count ratio < 40% or > 75%

Neutrophil count ratio abnormality did not differentiate between survival and mortality groups. Meanwhile, body temperature did not have discriminant potency, either. However, the WBC count in 7-day mortality group was much higher than other groups. Surgery patient’s population in 7-day mortality group presented to be the lowest among all groups, as shown in Table 1, indicating a surgical background may refer a better outcome in critical patients.

### Infection and microorganisms profile

Among total analyzed patients, 280 were detected with infection, including bacteria (total 301 isolates, with 275 gram-negative and 26 gram-positive isolates), fungi (69 isolates), virus (3 isolates), anaerobe (1 isolate) or tuberculosis (1 isolate). On the other hand, 97 cases (survival: 77 cases, 28-day mortality: 14 cases, 7-day mortality: 6 cases) were showed with mixed infection with multiple microorganisms, and other 148 cases (survival: 116 cases, 28-day mortality: 14 cases, 7-day mortality: 18 cases) were not infected. According to infection sites, the patients were categorized with pneumonia (208 cases), bacteremia (19 cases), peritonitis (23 cases), intra-cranial infection (15 cases), and other infections (15 cases). Characteristics of different infection sites were recorded in Table 2. The specific microorganism profile for each group was displayed in supplementary Table 1.

**Table 2:** Characteristics of infected and non-infected patients

	Non-infective ***n*** = 148	Infective					
		Pneumonia ***n*** = 208	Bacteremia ***n*** = 19	Peritonitis ***n*** = 23	Intra-cranial Infection ***n*** = 15	Other Infections n = 15	Total ***n*** = 280
**Age (year)**	67.6 ± 17.7	70.7 ± 18.3	68.5 ± 19.3	63 ± 18.5	60.5 ± 14.7	79.5 ± 13.8^*^	69.9 ± 18.3
**Gender (M/F)**	81/67	157/51	11/8	14/9	11/4	11/4	204/76
**Temperature abnormalities, n, (%)**	58 (39.2)	107 (51.4)^*^	13 (68.4)^*^	14 (60.9)	7 (46.7)	7 (46.7)	147 (52.5)^*^
**WBC abnormalities, n, (%)**	37 (25.0)	62 (29.8)	8 (42.1)	11 (47.8)^*^	8 (53.3)^*^	3 (20.0)	92 (32.9)
**NE abnormalities, n, (%)**	105 (70.9)	141 (67.8)	10 (52.6)	23 (100)^**^	13 (86.7)	9 (60.0)	196 (70.0)
**APACHE II score**	18.3 ± 5.2	21.3 ± 6.1^***^	21.5 ± 8.4^*^	20.5 ± 6.9	18.2 ± 6.2	21.6 ± 4.3^*^	21.1 ± 6.2^***^
**NLCR**	11.7 ± 13.5	11.3 ± 11.7	9.8 ± 8.5	17 ± 31.1	14.9 ± 11.4	10.8 ± 11.1	11.9 ± 14.1
**PCT (ng/ml)**	0.9 ± 2.5	4.3 ± 14.6^*^	5.3 ± 10.1	21.3 ± 35.1^***^	0.8 ± 1.3	1 ± 1.3	5.4 ± 16.9^***^
**CRP (mg/ml)**	46.6 ± 52.1	72 ± 78.5^*^	42.9 ± 40.9	117.4 ± 99.9^***^	70.8 ± 64.9	59.5 ± 68.6	73.4 ± 78.7^***^
**Blood lactate (mmol/l)**	1.4 ± 1.3	1.6 ± 1.1	2.4 ± 3.2	2 ± 1.1	1.6 ± 0.9	1.7 ± 0.9	1.8 ± 1.3^*^
**Surgery, n, (%)**	41 (27.7)	47 (22.6)	7 (36.8)	12 (52.2)^**^	11 (73.7)^***^	0 (0)^*^	77 (27.5)

Data were expressed as number (percentage) of patients or mean ± standard deviation. *p < 0.05,**p < 0.01, ***p < 0.001 vs non-infective group. Temperature abnormality: temperature < 36 °C or > 38 °C; WBC abnormalities: WBC < 4 × 10 ^ 9 / L or > 12 × 10 ^ 9 / L; NE abnormality: neutrophil count ratio < 40% or > 75%

### Co-morbid conditions

The incidence of cardiovascular co-morbid conditions on admission to the ICU was higher in patients of both 28- and 7-day mortality groups than in survival group, indicating that cardiovascular disease background could be a major risk factor for negative outcomes. On the other hand, the incidence of malignancies was much lower in mortality groups than that of survival group. Apart from this, the surgery operation incidence in survival group was much higher than that of 7-day mortality group, as described in Table 1. Other co-morbid diseases include diabetes mellitus, hypertension, COPD, liver cirrhosis and renal failure, which did not differentiate between groups. Details were presented in supplementary Table 2.

### Diagnostic character of APACHE II and biomarkers

APACHE II, one of several ICU scoring systems, is a severity-of-disease classification system. It is applied within 24 h of admission of a patient to an ICU. Higher APACHE II score negatively correlates with survival rate. In our study, we applied APACHE II to be the comparable reference for the analyzed biomarkers.

**Fig. 2:**
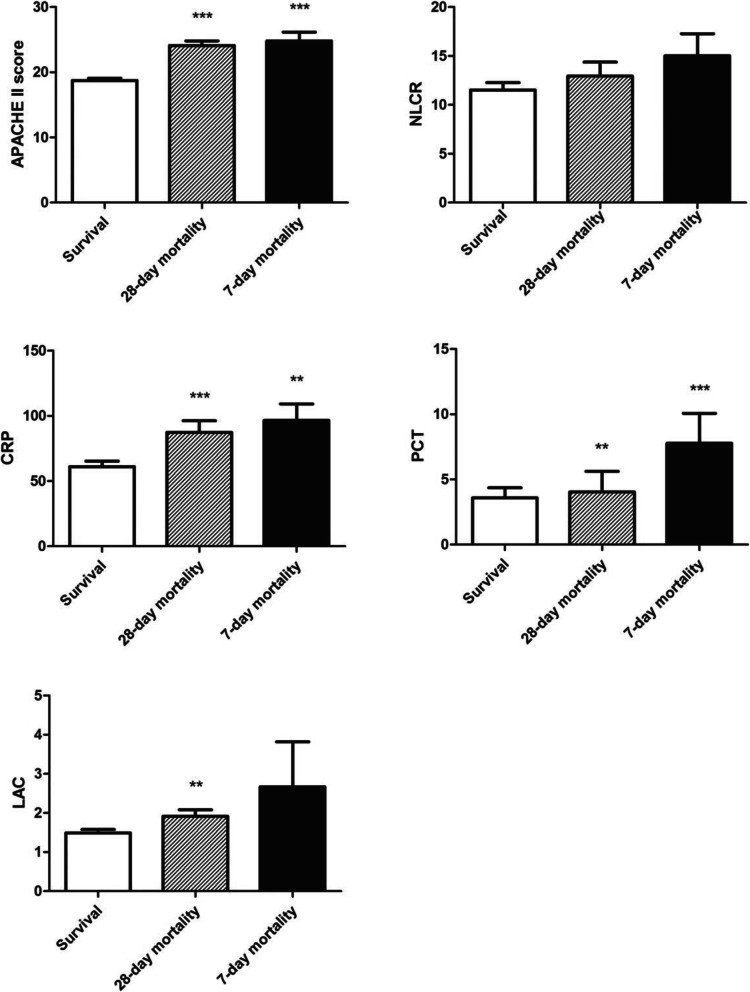
APACHE II scores and biomarker levels of NLCR, CRP, PCT, and LAC of survival, 28-day mortality, and 7-day mortality group. **p < 0.01 vs survival, ***p < 0.001 vs survival

NLCR, APACHE II score and other biomarker levels of survival, 28- and 7-day mortality groups were showed in Fig. 2. By studying cohort of 428 critical patients, the APACHE II score at admission in mortality groups were much higher than that of survivors. Both CRP and PCT levels of mortality groups were significantly elevated than those of survival groups (Table 1, Fig. 2). Regarding to LAC, only 28-day mortality group levels were higher than that of survivals, but not 7-day mortality group levels. This is probably because by relatively large deviation and small count of group population. Of note, NLCR did not vary among survivals and non-survival patients (Table 1, Fig. 2).

### Discriminant performance of APACHE II and biomarkers

The sensitivity of NLCR, CRP, PCT, LAC and APACHE II score to predict 28- and 7-day mortality was presented in Fig. 2. With group comparison, we found that PCT, CRP and APACHE II score were all discriminant between survival and mortality groups, but not NLCR. Among all biomarkers, both PCT and CRP showed greater differential potency than others with lowest *p* value at comparison of the survival and mortality groups. On the other hand, LAC showed less differential potency among all groups (Fig. 2).

**Fig. 3:**
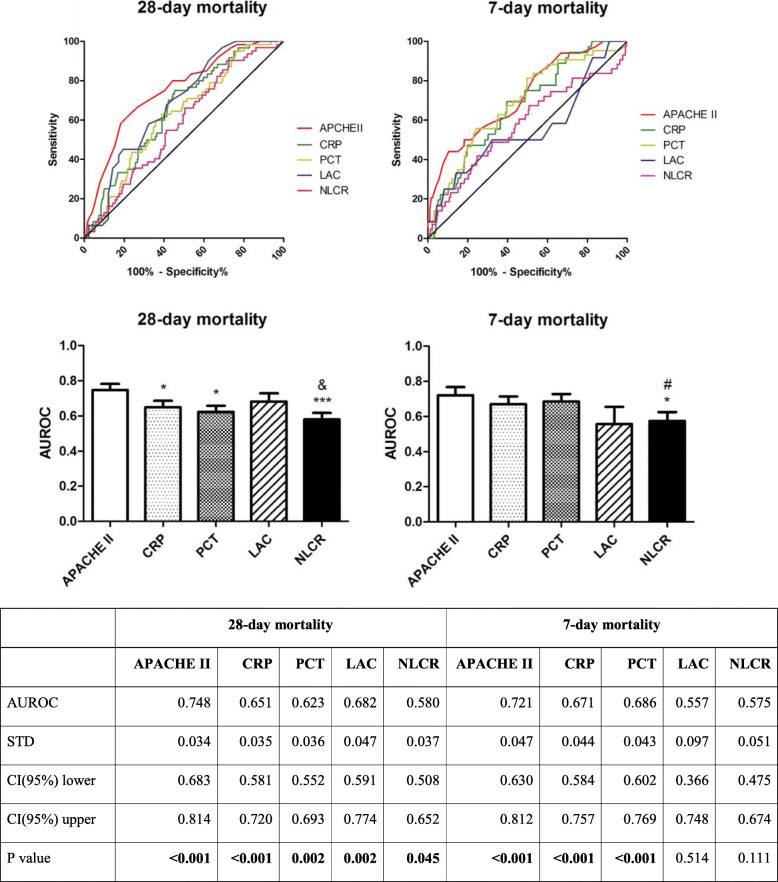
Receiver operating characteristic curves and AUROCs of markers for predicting overall 28-day and 7-day mortality. *P < 0.05, ***P < 0.001 compared with APACHE II, ^&^*P* < 0.05 compared with LAC, ^#^*P* < 0.05 compared with PCT. The table below showed the area under the ROC curve value with STD, 95% confidence intervals and *P* values versus survival group

According to area under ROC curves analysis, the AUROCs of NLCR were 0.580 and 0.575 (with *P* values of 0.045 and 0.111) for 28- and 7-day mortality group, respectively. This suggests that NLCR is not a powerful discriminator to predict mortality. On the other hand, not only APACHE II score, but also CRP and PCT had much higher levels of AUROCs than NLCR (Fig. 3).

To identify 28-day mortality, APACHE II score presented highest AUROC than all the biomarkers, while PCT and CRP presented to be less potent biomarkers. LAC had relatively higher levels of AUROC, which made it a better indicator to predict 28-day mortality. Moreover, LAC had the lowest value of prediction, while APACHE II score remained the highest value of predicting 7-day mortality. Compared to PCT and CRP, NLCR was a relatively weak biomarker to predict both short and long term mortality according to AUROC levels (data shown in Fig. 3).

**Fig. 4:**
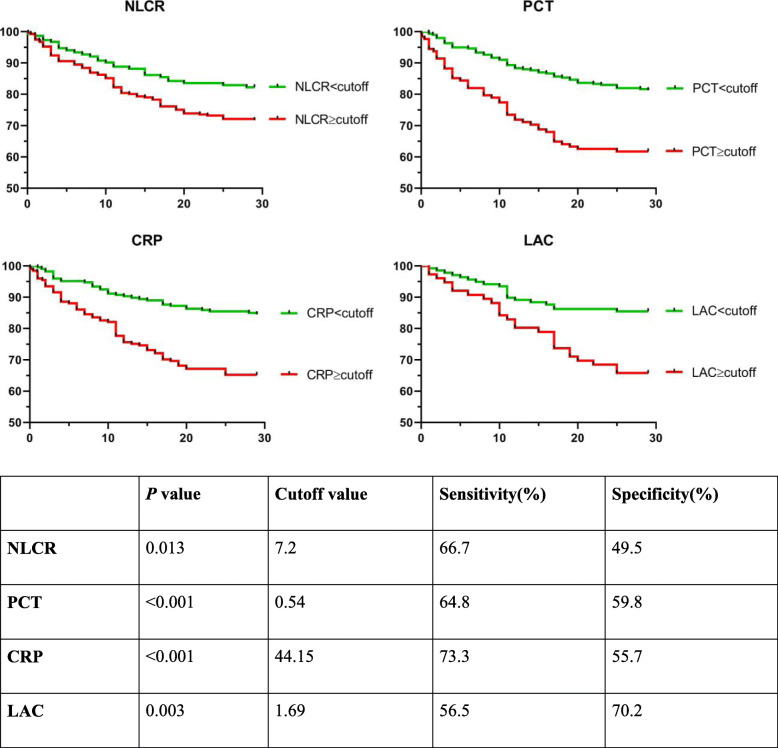
Survival curves of biomarkers divided by respective cutoff values. The table below showed the P values, cutoff values, sensitivity and specificity

In regard to survival analysis, all the biomarkers selected in this study presented good potential to predict outcomes of critical patients. We calculated the cutoff values for each biomarker and graphed the survival curves accordingly. The results showed both PCT and CRP had higher sensitivity and specificity than NLCR and LAC with *p* values lower than 0.001. Compared to PCT and CRP, NLCR failed to indicate prognosis of critical patients (Fig. 4).

## Discussion

In this study, we compared the discriminatory ability of NLCR with CRP, PCT and APACHE II score on prediction of critical illness. The results indicates that PCT, CRP and APACHE II score were all discriminant between survival and mortality group, not NLCR. The ROC analysis also revealed APACHE II score, CRP, PCT and LAC had higher levels of AUROCs than NLCR, which made NLCR a relatively weak predictor of mortality. For prediction of 28-day mortality, APACHE II score and LAC presented higher efficacy than all other markers. Meanwhile, for prediction of 7-day mortality, both CRP and PCT displayed comparable efficacy as well as APACHE II score.

NLCR, as a relatively new biomarker, numerous groups have demonstrated it was able to predict outcomes of various oncology patients and served as prognostic pre-operatively in patients with colorectal cancer [1, 15–18]. In addition, there are also current studies showing an association between NLCR and the prognosis and mortality in infectious diseases. Previous investigations had already presented that NLCR was a more sensitive parameter in the prediction of appendicitis [19]. Zahorec and colleagues have observed lymphocytopenia in ICU patients following major surgery and sepsis, and noticed higher levels of NLCR related with severity of the clinical courses [1]. Huang and colleagues analyzed previous studies and concluded that the NLCR was associated with the prognosis of sepsis and that a higher NLCR indicate unfavorable prognosis [20]. In this study, we demonstrated that NLCR did not have high discriminant ability to predict outcomes of critical illness. Compared to conventional biomarkers and APACHE II score, AUROCs of NLCR did not show advantages of differentiation but borderline predictive capability of 28-day mortality. On the other hand, not only APACHE II score, but also CRP and PCT showed better potential of prognostic value on mortality outcomes of critical illness.

Regarding to conventional biomarkers, Wyllie and colleagues have determined that CRP alone could not precisely predict bacterial infection than lymphocytopenia alone or a combination of lymphocytopenia and neutrophilia [21]. Although procalcitonin has been evaluated and proved to be prognostic with critical illness, especially of septic inflammation, its implementation has been hampered due to the high costs and lacking of accessibility in developing countries.

Elevated LAC is often considered a marker of circulatory ischemia and hypoxia and is also directly related to the prognosis of sepsis. In our study, LAC levels were less powerful in predicting 7-day mortality than 28-day mortality. These results were in accordance with some of previous conclusions [6, 22], but contradicted with others [5]. This may cause by insufficient population or tumor background, which may not be able to applied to patients with inflammatory background. Our study enrolled 428 cases into final analysis, which presented a relatively large study population and hence improved its reliability.

Meanwhile, most recent investigations demonstrated that NLCR, a simple and easily obtainable marker, had higher predictive value in bacterial infection, and can be integrated into daily practice without extra costs [5]. With these characteristics, NLCR was even suggested to be widely applied to the surveillance protocols of clinical scenarios especially in developing countries. However, based on AUROCs calculation and comparison, NLCR was proved to be less valuable than any of the above-mentioned conventional biomarkers and APACHE II score to predict prognosis or to evaluate the severity of a disease. Moreover, NLCR levels did not distinguish between survival and mortality groups, which suggested it may not capable to be a reliable indicator to evaluate severity or to predict prognosis of critical illness.

## Conclusion

NLCR as a non-specific biomarker, was associated with both 7- and 28-day mortality in adult critical patients. However, both CRP and PCT were more sensitive and specific to predict prognosis in critical ill patients. Compared to traditional predictive indicators, NLCR shows no advantages over PCT, CRP, and APACHE II score. Thus, NLCR could not be an ideal substitute to conventional markers in evaluation of severity of critical illness.

### Limitations of this study

Indeed, the present study does have several limitations. First, although this study involves a relatively large population, it is a retrospective study, thus the potential of this study is to be further determined by prospective researches in much larger population in other centers. Second, this study generally compared the potential of NLCR and other traditional inflammatory markers such as CRP, PCT, white blood cell count, neutrophil count and APACHE II severity score, but not the more potentially predictive markers. Further studies should be conducted to evaluate such predictive potentials and compare them with those short- and long-term bio-reactive proteins, such as acute phase proteins in this context. Third, this study did not separately demonstrate relationships between NLCR and risk of death in infected patients. At this point, it may contradict with the conclusions of other literatures.

## Supplementary Information

**Additional file 1: Table S1.** Microorganisms isolated from the patients in the study cohort. **Table S2.** Coexisting disease of the study population stratified by survival and mortality

## Data Availability

The data-sets used and/or analyzed during the current study are available from the corresponding author on reasonable request.

## Abbreviations

APACHE II: Acute Physiology and Chronic Health Evaluation II
AUROC: Area under the ROC curve
CI: Confidence interval
CRP: C-reactive protein
ICU: Intensive care unit
IRB: Institutional review boards
LAC: Lactate
NE: Neutrophil
NLCR: Neutrophil-lymphocyte count ratio
PCT: Procalcitonin
ROC: Receiver operating characteristic
WBC: White blood cell
