# Supplementary material for: Prognostic value of neutrophil- lymphocyte count ratio (NLCR) among adult ICU patients in comparison to APACHE II score and conventional inflammatory markers: a multi center retrospective cohort study
Source: BMC Emerg Med. 2021 Feb 23;21:24. doi: 10.1186/s12873-021-00418-2 (PMC7903782; doi:10.1186/s12873-021-00418-2)
Supplement: Supplementary file 1 — Additional file 1: Table S1. Microorganisms isolated from the patients in the study cohort. Table S2. Coexisting disease of the study population stratified by survival and mortality [file 12873_2021_418_MOESM1_ESM.docx]

**Supplementary data**

Table 1. Microorganisms isolated from the patients in the study cohort

|  | Survival  n=323 | **28-d mortality**  **n = 62** | **7-d mortality**  **n = 43** |
| --- | --- | --- | --- |
| Gram-Negative isolates (n, %) | **216 (66.9%)** | **39 (62.9%)** | **20 (46.5%)*** |
| Acinetobacter baumannii | 75 (23.2%) | 19 (30.6%) | 13 (30.2%) |
| Klebsiella spp. | 44 (13.6%) | 14 (22.6%) | 3 (7.0%) |
| Pseudomonas spp. | 43 (13.3%) | 5 (8.1%) | 2 (4.7%) |
| Enterobacter spp. | 21 (6.5%) | 1 (1.6%) | 1 (2.3%) |
| S. maltophilia | 14 (4.3%) | 0 (0) | 1 (2.3%) |
| Other | 19 (5.9%) | 0 (0) | 0 (0) |
| Gram-positive isolates (n, %) | **13 (4.0%)** | **9 (14.5%)**** | **4 (9.3%)** |
| S.Aureus | 6 (1.9%) | 6 (9.7%)** | 1 (2.3%) |
| MRSA | 2 (0.6%) | 1 (1.6%) | 1 (2.3%) |
| Streptococcus spp. | 2 (0.6%) | 0 (0) | 1 (2.3%) |
| Enterococcus spp. | 2 (0.6%) | 1 (1.6%) | 1 (2.3%) |
| Other | 1 (0.3%) | 1 (1.6%) | 0 (0) |
| Fungi isolates (n, %) | **52 (16.1%)** | **10 (16.1%)** | **7 (16.3%)** |
| Candida albicans | 35 (10.8%) | 8 (12.9%) | 2 (4.7%) |
| Candida glabrada | 9 (2.8%) | 1 (1.6%) | 1 (2.3%) |
| Candida tropicalis | 5 (1.5%) | 1 (1.6%) | 1 (2.3%) |
| Other | 3 (0.9%) | 0 (0) | 3 (%)* |
| Virus isolates (n, %) | **2 (0.6%)** | **1 (1.6%)** | **0 (0)** |
| Anaerobes isolates (n, %) | **1 (0.3%)** | **0 (0)** | **0 (0)** |
| Tuberculosis isolates (n, %) | **0 (0)** | **0 (0)** | 1 (2.3%) |

Data presented as number (percentage of group population) of isolates, instead of number of patients. *p < 0.05, **p < 0.01 vs survival group.

Table 2 Coexisting disease of the study population stratified by survival and mortality

|  | **Survival**  **n = 323** | **28-d mortality**  **n = 62** | **7-d mortality**  **n = 43** |
| --- | --- | --- | --- |
| Diabetes mellitus | 3 (0.9%) | 0 (0) | 0 (0) |
| Cardiovascular disease | 18 (5.6%) | 4 (6.5%) | 10 (23.3%)*** |
| Hypertension | 4 (1.2%) | 0 (0) | 0 (0) |
| Malignancies | 29 (9.0%) | 1 (1.6%)** | 4 (9.3%) |
| COPD | 8 (2.5%) | 4 (6.5%) | 1 (2.3%) |
| Liver cirrhosis | 0 (0) | 0 (0) | 0 (0) |
| Renal failure | 6 (1.9%) | 4 (6.5%) | 2 (4.7%) |

Data were expressed as number (percentage of current group), ** p < 0.01, *** p < 0.001 vs survival group. COPD, chronic obstructive pulmonary disease.
